# A Planar Multi-Inertial Navigation Strategy for Autonomous Systems for Signal-Variable Environments

**DOI:** 10.3390/s24041064

**Published:** 2024-02-06

**Authors:** Wenbin Dong, Cheng Lu, Le Bao, Wenqi Li, Kyoosik Shin, Changsoo Han

**Affiliations:** 1 Department of Mechatronics Engineering, Hanyang University, Ansan 15588, Republic of Korea; dongwb@ahstu.edu.cn (W.D.); baole@hanyang.ac.kr (L.B.); liwenqi@hanyang.ac.kr (W.L.); cshan@hanyang.ac.kr (C.H.); 2 School of Mechanical Engineering, Anhui Science and Technology University, Chuzhou 233100, China; luc@ahstu.edu.cn

**Keywords:** mobile robot, EKF, INS, localization, autonomous navigation

## Abstract

The challenge of precise dynamic positioning for mobile robots is addressed through the development of a multi-inertial navigation system (M-INSs). The inherent cumulative sensor errors prevalent in traditional single inertial navigation systems (INSs) under dynamic conditions are mitigated by a novel algorithm, integrating multiple INS units in a predefined planar configuration, utilizing fixed distances between the units as invariant constraints. An extended Kalman filter (EKF) is employed to significantly enhance the positioning accuracy. Dynamic experimental validation of the proposed 3INS EKF algorithm reveals a marked improvement over individual INS units, with the positioning errors reduced and stability increased, resulting in an average accuracy enhancement rate exceeding 60%. This advancement is particularly critical for mobile robot applications that demand high precision, such as autonomous driving and disaster search and rescue. The findings from this study not only demonstrate the potential of M-INSs to improve dynamic positioning accuracy but also to provide a new research direction for future advancements in robotic navigation systems.

## 1. Introduction

The field of autonomous navigation has seen diverse advancements in localization techniques, each tailored for specific challenges, particularly in environments with unstable signal strengths and indoor settings.

Recent advancements in GNSS/INS integration have notably improved localization in challenging environments. Cao S. et al. [1] introduced GVINS, a system that integrates GNSS, as well as visual and inertial data for accurate state estimation. Building upon this, Pan C. et al. [2] introduced a system that merges monocular visual-inertial odometry with PPP for better urban environment navigation. Additionally, Farhangian F. et al. [3] proposed an alignment method for SINS, using LEO satellite Doppler shift measurements, enhancing the accuracy where GNSS is unavailable. However, their effectiveness is challenged in environments lacking GNSS signal, such as in indoor settings. The research by Jiang C. et al. [4] developed a mixed norm-based data fusion algorithm for GNSS/INS-integrated navigation systems. Sensor fusion technologies, as discussed in studies by Fayyad J. et al. [5], Debeunne C. et al. [6], and Liu X. et al. [7], are pivotal in improving both accuracy and reliability. Chen J et al. [8] explore the fusion of inertial navigation with Bluetooth for indoor localization, addressing the challenges in GPS-denied environments. Indoor localization poses unique challenges, addressed by UWB and vision-based systems in research by Chen Y. Y. et al. [9], Hu J. et al. [10], and Masiero A. et al. [11]. These studies highlight the potentials and limitations of UWB and computer vision in complex indoor environments. The real-time localization requirements in dynamic environments, such as construction sites, are discussed by Jeelani I. et al. [12] and Xu Z. et al. [13], emphasizing the need for accurate and responsive navigation systems. The integration of vision and LiDAR technologies in autonomous systems, as reviewed by Chen X. et al. [14] and Caballero F. et al. [15], showcases their application in scenarios where high precision is required. However, these technologies often face challenges in adverse weather conditions or in environments with limited spatial features. The importance of anomaly detection in dynamic environments is highlighted in the work of Mishra P. et al. [16]. Navigation in agricultural settings, as explored by Zhao W. et al. [17], poses its own set of challenges, particularly in open-field conditions where signal variability can impact system performance.

In response to the challenges in autonomous navigation, particularly in indoor and signal-variable environments, our research introduces a multi-INS-based approach. This method leverages the inherent stability of INSs and integrates multiple units to enhance localization accuracy and reliability. By reducing the dependence on external signals, our approach offers a robust solution, particularly for environments where traditional systems falter. This advancement is crucial for applications requiring high precision and operational consistency in diverse settings, marking a significant step forward in the field of autonomous navigation.

## 2. Methods

### 2.1. Theory of Multi-Inertial Navigation System (M-INS) Positioning

**Figure 1:**
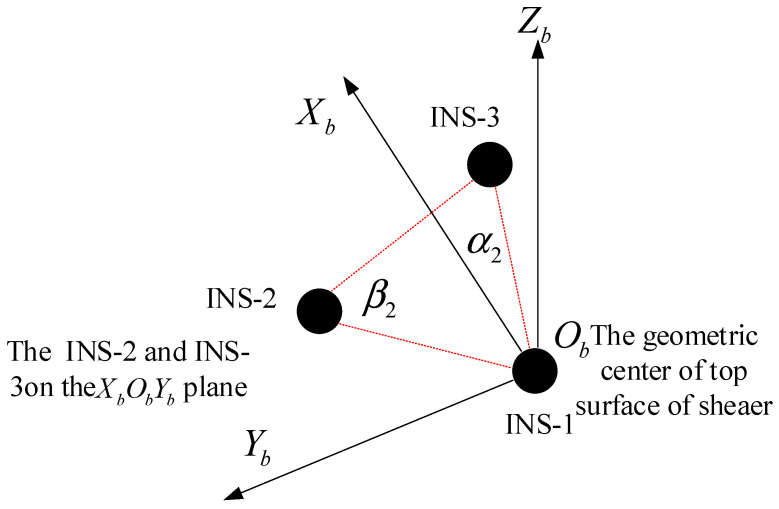
Three INS positioning working principle diagram.

Positioning within the realm of autonomous navigation systems is fundamentally enhanced by the multi-inertial navigation system (M-INS) approach. This system, depicted in Figure 1, represents a sophisticated configuration that leverages the synergistic capabilities of three spatially distributed INS units. These units are arranged in a co-planar fashion, with one unit designated as the origin of the local coordinate system, while the others are aligned along the orthogonal *X* and *Y* axes. The rationale behind such an arrangement is rooted in the principle of geometrical constancy and spatial redundancy, which is key to enhancing the positioning accuracy without reliance on external signals.

The inherent strength of the M-INS configuration lies in its structural resilience to common navigational errors. Unlike solitary INS setups that are prone to cumulative errors over time, the M-INS framework utilizes the fixed relative distances between the INS units—labeled r_12_, r_23_, and r_13_—as invariant constraints, which serve as a baseline for the system’s spatial orientation and positioning. This rigidity in spatial relationships ensures that even in the absence of external reference points, the M-INS can maintain a high degree of accuracy through internal checks and balances. Attitude information, encompassing the heading, pitch, and roll of each INS, is foundational to the M-INS operation. These orientations are integral to defining the relative geometrical positioning of the units, providing the necessary data to establish the orientation matrix for the system. This orientation matrix, built upon the attitude data, translates the inertial measurements into a comprehensive navigational solution within the local coordinate frame, establishing the basis for subsequent motion analysis and trajectory prediction.

**Figure 2:**
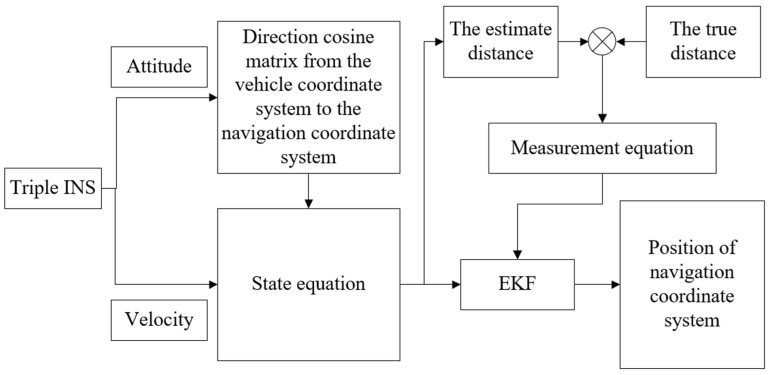
The composition and workflow of M-INSs.

To encapsulate the process, Figure 2 visually represents the workflow from capturing the attitude and velocity data from the triple INS setup, through the state equation that encompasses the direction cosine matrix conversion, to the measurement equation where the estimated distances are refined by the EKF, culminating in the final position within the navigation coordinate system. This streamlined process underscores the algorithm’s ability to process and integrate complex sensor data to deliver precise positional information.

### 2.2. M-INS’s Algorithm Depending on EKF

In a multi-inertial navigation system (M-INS), three INS units work in concert to collect attitude and velocity information. This data undergoes a transformation through direction cosine matrices (DCMs) to align it with the navigation coordinate system. Position updates for the INSs are then calculated using the increments derived from these transformations, as expressed in Equation (1):

(1) $$X_{t}=A_{t}X_{t-1}+B_{t}v_{t}+W_{t}$$

here, *B_t_* represents the DCM, *X_t−1_* is the state vector at the previous time step, *v_t_* is the velocity vector, and *W_t_* is the process noise. The state vector *X* includes the coordinates (*xi*, *yi*, *zi*) of each INS unit (*i* = 1 to 3) and the velocity vector v contains the velocities (*v_xi_*, *v_yi_*, *v_zi_*) for each unit (*i* = 1 to 3) along the three axes as Equations (2)–(5):

(2) $$X={\left[\begin{array}{ccccccccc} x_{1} & y_{1} & z_{1} & x_{2} & y_{2} & z_{2} & x_{3} & y_{3} & z_{3} \end{array}\right]}^{T}$$

(3) $$v={\left[\begin{array}{ccccccccc} v_{x1} & v_{y1} & v_{z1} & v_{x2} & v_{y2} & v_{z2} & v_{x3} & v_{y3} & v_{z3} \end{array}\right]}^{T}$$

(4) $$B_{i}=\left[\begin{array}{ccc} \cos\gamma_{i}\cos\varphi_{i}+\sin\gamma_{i}\sin\theta_{i}\sin\varphi_{i} & \cos\theta_{i}\sin\varphi_{i} & \sin\gamma_{i}\cos\varphi_{i}-\cos\gamma_{i}\sin\theta_{i}\sin\varphi_{i}\\ -\cos\gamma_{i}\sin\varphi_{i}+\sin\gamma_{i}\sin\theta_{i}\cos\varphi_{i} & \cos\theta_{i}\cos\varphi_{i} & -\sin\gamma_{i}\sin\varphi_{i}+\cos\gamma_{i}\sin\theta_{i}\cos\varphi_{i}\\ -\sin\gamma_{i}\cos\theta_{i} & \sin\theta_{i} & \cos\gamma_{i}\cos\theta_{i} \end{array}\right]$$

(5) $$B=\left[\begin{array}{ccc} B_{1} & 0 & 0\\ 0 & B_{2} & 0\\ 0 & 0 & B_{3} \end{array}\right]$$

here, *B_i_* is the DCM of each INS. The measurement model is depicted in Equation (6), with *V* as the noise, *R* as the actual distances between the INS units, and *r_ij_* representing the measured distances obtained through the INS.

(6) $$z={\left[\begin{array}{ccc} R_{12}-r_{12} & R_{23}-r_{23} & R_{13}-r_{13} \end{array}\right]}^{T}+V$$

The measurement distances r are calculated as shown in Equation (7).

(7) $$\left[\begin{array}{c} r_{12}\\ r_{23}\\ r_{13} \end{array}\right]=\left[\begin{array}{c} \sqrt{{\left(x_{1}-x_{2}\right)}^{2}+{\left(y_{1}-y_{2}\right)}^{2}+{\left(z_{1}-z_{2}\right)}^{2}}\\ \sqrt{{\left(x_{2}-x_{3}\right)}^{2}+{\left(y_{2}-y_{3}\right)}^{2}+{\left(z_{2}-z_{3}\right)}^{2}}\\ \sqrt{{\left(x_{1}-x_{3}\right)}^{2}+{\left(y_{1}-y_{3}\right)}^{2}+{\left(z_{1}-z_{3}\right)}^{2}} \end{array}\right]$$

A first-order Taylor expansion of Equation (6) yields the Jacobian matrix *H* of the measurement model, outlined in Equation (8).

(8) $$H=\left[\begin{array}{ccccccccc} \frac{x_{2}-x_{1}}{r_{12}} & \frac{y_{2}-y_{1}}{r_{12}} & \frac{z_{2}-z_{1}}{r_{12}} & \frac{x_{1}-x_{2}}{r_{12}} & \frac{y_{1}-y_{2}}{r_{12}} & \frac{z_{1}-z_{2}}{r_{12}} & 0 & 0 & 0\\ 0 & 0 & 0 & \frac{x_{3}-x_{2}}{r_{23}} & \frac{y_{3}-y_{2}}{r_{23}} & \frac{z_{3}-z_{2}}{r_{23}} & \frac{x_{2}-x_{3}}{r_{23}} & \frac{y_{2}-y_{3}}{r_{23}} & \frac{z_{2}-z_{3}}{r_{23}}\\ \frac{x_{3}-x_{1}}{r_{13}} & \frac{y_{3}-y_{1}}{r_{13}} & \frac{z_{3}-z_{1}}{r_{13}} & 0 & 0 & 0 & \frac{x_{1}-x_{3}}{r_{13}} & \frac{y_{1}-y_{3}}{r_{13}} & \frac{z_{1}-z_{3}}{r_{13}} \end{array}\right]$$

Building upon the state and measurement equations, the M-INS employs an extended Kalman filter model for indoor navigation:

Prediction Step: The mean state vector prediction for the INS coordinates in the navigation frame is constructed using Equation (1), forming a priori estimate equations as:

(9) $${\hat{X}}_{t}=A_{t}X_{t-1}+B_{t}v_{t}$$

where ${\hat{X}}_{t}$ denotes the a priori estimate of the INS position at time *t*.

The prior covariance matrix is updated as:

(10) $${\hat{\Sigma }}_{t}=G_{t}\Sigma_{t-1}G_{t}^{T}+R_{t}$$

In the equation, ${\hat{\Sigma }}_{t}$ is the prior covariance at time t, $\Sigma_{t-1}$ is the posterior covariance at the previous time step, and *R_t_* represents the covariance of the process noise.

Kalman gain update: The Kalman gain update process allocates weight between the measured INS positions in the navigation frame and the a priori estimates, updating the posterior estimates as:

(11) $$K_{t}={\hat{\Sigma }}_{t}H_{t}^{T}{\left(H_{t}{\hat{\Sigma }}_{t}H_{t}^{T}+Q_{t}\right)}^{-1}$$

where *K_t_* is the Kalman gain at time *t*, and *Q_t_* is the covariance of the measurement noise *V*.

Update Step: The posterior INS positions in the navigation frame are determined by combining the a priori estimates, the measurements, and the Kalman gain:

(12) $$X_{t}={\hat{X}}_{t}+K_{t}(z_{t}-H_{t}{\hat{X}}_{t})$$

(13) $$\Sigma_{t}=(I-K_{t}H_{t}){\hat{\Sigma }}_{t}$$

where $\Sigma_{t}$ is the posterior covariance at time *t*. Equations (9)–(13) encompass the components of the EKF model based on three INS units.

**Figure 3:**
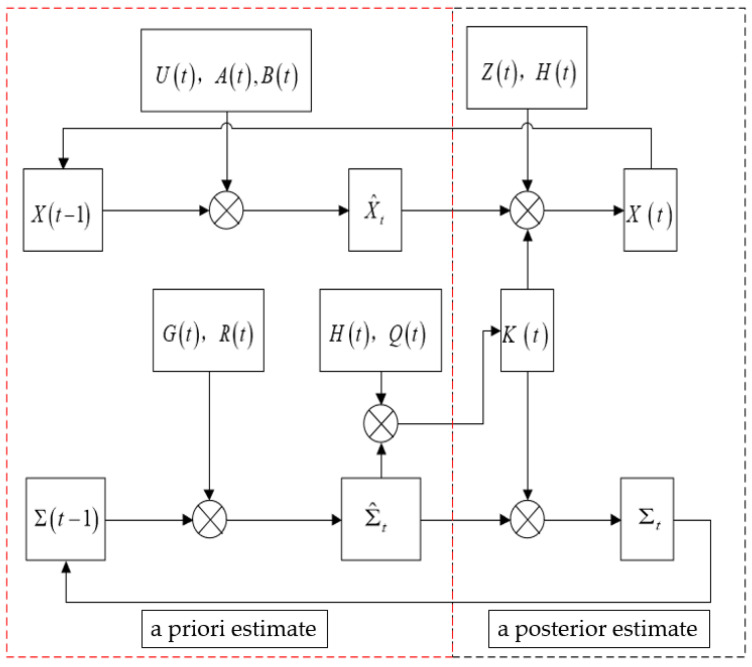
Three INSs’ EKF algorithm flow chart.

The algorithmic flow of the M-INSs based on EKF is a sequential process that ensures the fusion of data from each INS, to achieve an accurate and reliable navigation solution. This approach significantly mitigates the inherent errors of the individual INS units, leveraging the EKF’s ability to filter and integrate the noisy sensor data effectively. The filtering process is shown in Figure 3.

## 3. M-INS Mobile Robot Positioning Simulation and Experimental

### 3.1. Simulation

**Figure 4:**
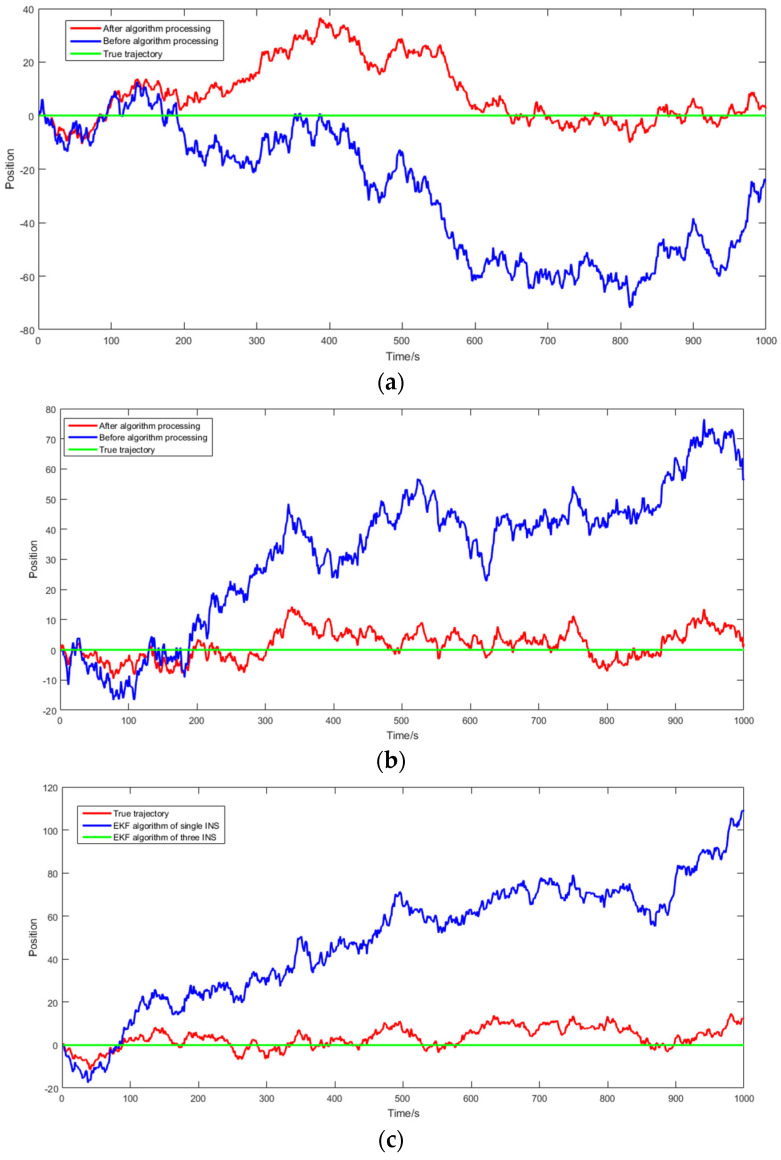
Simulation of the robot position errors for the *x*–direction in (**a**), *y*–direction in (**b**), and the *z*–direction in (**c**).

To validate the effectiveness of the multi-inertial navigation system (M-INS) employing an extended Kalman filter (EKF), a series of simulations were conducted. Utilizing MATLAB, the simulation environment replicated the fixed-point static of an object equipped with INSs and applied the EKF algorithm to process the INS data. The aim was to compare the trajectory estimation accuracy of a single INS against that of the M-INS configuration. To better validate the M-INS algorithm, a significant level of noise was introduced, with a standard deviation of five. The simulation was conducted over a period of *t* = 1000 s, and the results are presented in Figure 4.

During the simulation, the multi-inertial navigation system (M-INS) algorithm significantly enhanced the positional accuracy along the *x*, *y*, and *z* axes. The *x*–directional data, initially marked by large fluctuations, showed a notable alignment with the true trajectory after applying the M-INS algorithm. Similar improvements were observed in the *y*–direction, where the erratic trends were markedly subdued, indicating a stable and consistent trajectory. The most profound correction was evident in the *z*–direction, where the M-INS algorithm effectively minimized vertical drift, maintaining a trajectory that closely paralleled the actual path. Cumulatively, these results underscore the M-INS algorithm’s capability to substantially mitigate navigational errors, proving its efficacy for precise and reliable navigation across varied dimensions.

### 3.2. Experimental

#### 3.2.1. Experimental Device

**Figure 5:**
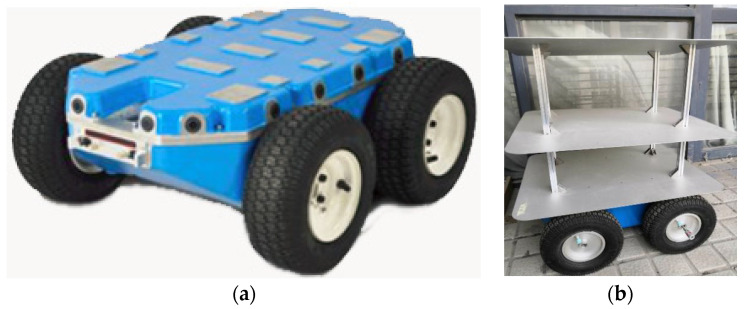
Mobile robot structure for moving chassis (**a**) and whole robot (**b**).

For the empirical examination of the M-INS enhanced by the EKF, the experimental setup involved a specifically designed mobile robot, modeled after the Navigator C2. This adaptable platform was equipped to carry a three-tiered rack structure, facilitating the installation of three INS units in a co-planar arrangement, as well as a GPS-RTK mobile station for ground truth data acquisition, as shown in Figure 5.

**Figure 6:**
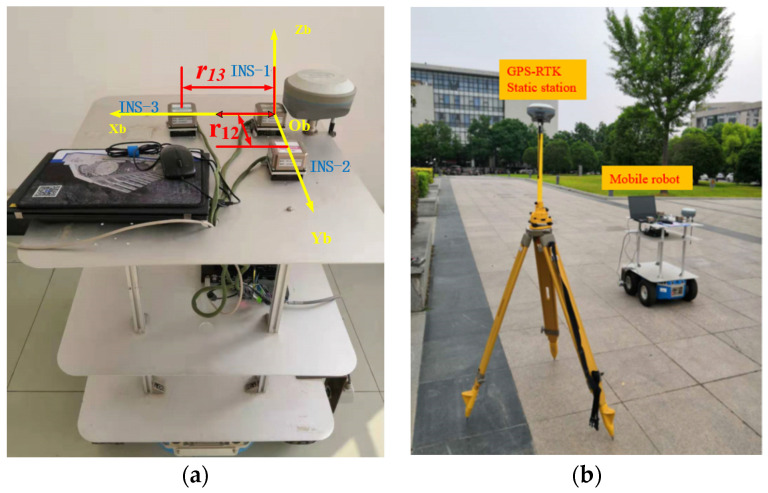
Experimental equipment in (**a**) and experimental site in (**b**).

The arrangement of the INS units was meticulously planned: INS-1 was established as the origin point of the coordinate system, with INS-2 and INS-3 placed along the *X* and *Y* axes, respectively, maintaining a fixed distance of 0.38 m from the origin (as depicted in Figure 6a). This configuration was critical to ensure the integrity of the inter-INS measurements and the overall efficacy of the M-INS. Figure 6b showcases the experimental apparatus in detail. It features the mobile robot with the INS units mounted on the tiers of the rack, alongside the GPS-RTK static station set up in an outdoor environment. The setup was designed to emulate real-world conditions, where the GPS-RTK station could provide a reliable reference trajectory, unimpeded by potential indoor signal interference. The INS employed in this study is an MEMS strapdown system, comprising tri-axial gyroscopes, accelerometers, and magnetometers. It features an RS422 communication interface with a baud rate of 230,400. The gyroscopes exhibit an RMS (root mean square) error of 0.3°/s, while the accelerometers demonstrate an RMS error of 400 μg.

#### 3.2.2. Result

**Figure 7:**
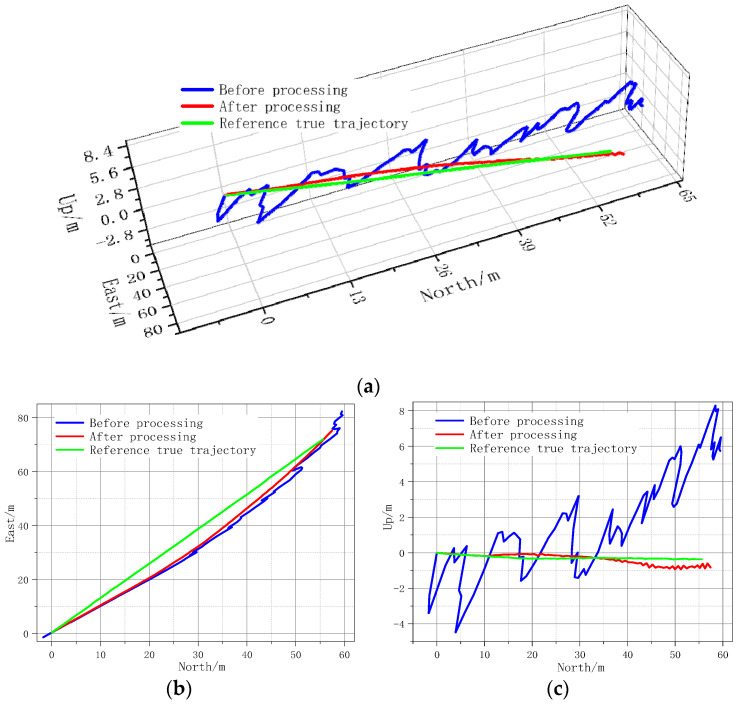
INS positioning trajectories (**a**) 3D space; (**b**) east-north plane; and (**c**) north-up plane.

The experimental evaluation meticulously examines the performance of the stand-alone INS operation against the composite M-INS algorithm enhanced by the EKF. This test posed a challenge to the algorithms, with GPS-RTK serving as the benchmark for the true trajectory. The findings, depicted in Figure 7, detail the trajectories in three-dimensional space and plot the positional errors across the east-north and north-up planes. In the east-north plane, the trajectory processed by the single INS algorithm shows substantial deviation, particularly in high-error conditions, leading to a potential divergence. The M-INS algorithm, however, maintains a consistently closer alignment with the true trajectory provided by the GPS-RTK reference, showcasing its robustness amidst significant noise.

**Figure 8:**
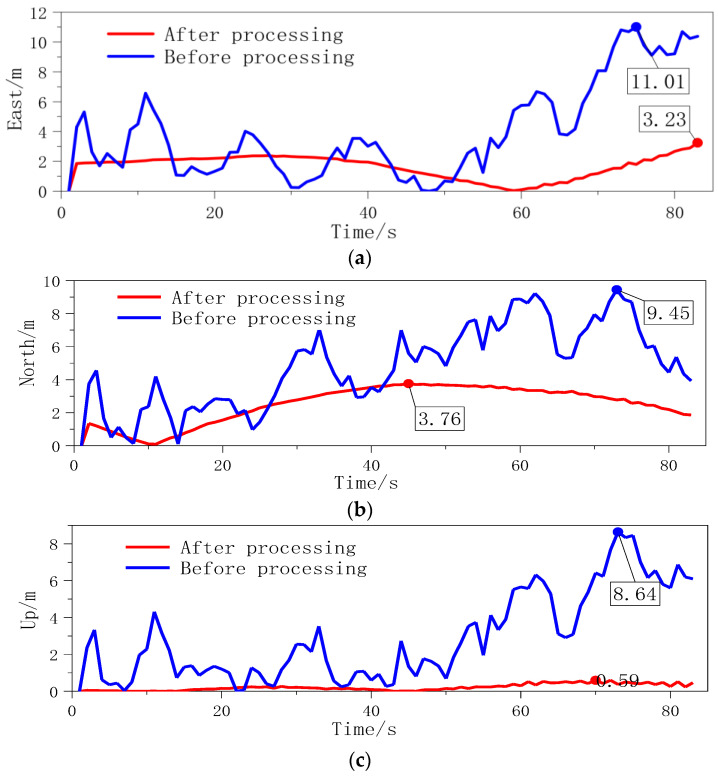
INS-1 error diagram (**a**) east error; (**b**) north error; and (**c**) up error.

In the north-up plane, the M-INS algorithm’s performance further validates its precision, with notably less vertical deviation, indicating an improved altitude estimation over the single INS algorithm. The trajectory comparison and error analysis are presented in Figure 8. The M-INS configuration exhibits a marked reduction in the maximum positional errors, bringing them down to 3.23 m, 3.76 m, and 0.60 m from the initial readings of 11.01 m, 9.45 m, and 8.64 m.

This improvement is clearly quantified in Table 1, which presents the spherical error probability (SEP) [18] results. Post processing, each INS unit sees an accuracy improvement exceeding 60%, demonstrating the efficacy of the M-INS’s error correction capability.

These results illustrate the M-INS’s superior performance in experimental conditions, with SEP values showing significant reductions from 7.23 m, 6.21 m, and 7.77 m to 2.29 m, 2.24 m, and 2.38 m, reflecting improvements of 68.33%, 63.93%, and 69.37%, respectively. The substantial enhancements in precision emphasize the potential of the M-INS system for high-accuracy applications and validate its utility in real-world navigational tasks, where accurate positional data is paramount.

## 4. Discussion

This study conducted an in-depth investigation into the positioning accuracy issues of multi-inertial navigation systems (MINSs). The MINS algorithm adopted in the research was able to significantly reduce the positioning deviations caused by the inherent errors of INSs. Using the sphere of probability method, it was observed that the positioning accuracy of each INS improved by more than 60%. By utilizing the known fixed distances between the three INS units as constraints, the precision of dynamic positioning was successfully enhanced, which is critical for robots in actual motion. Although the experimental results demonstrate an excellent performance of the algorithm in dynamic tests, several key issues still need attention. Firstly, there may be differences between the experimental environment and the real-world operational environment, hence further testing of the algorithm’s adaptability in more complex and variable real-world application scenarios is required. Secondly, the performance of the algorithm may be influenced by the quality and configuration of the sensors, necessitating validation across different levels of sensor hardware. Lastly, a more thorough analysis and optimization of the algorithm’s stability and robustness under nonlinear errors and extreme motion conditions are required.

## 5. Conclusions

The MINS-based extended Kalman filter algorithm proposed in this article has significantly improved the positioning accuracy of mobile robots under dynamic conditions. Experimental results clearly demonstrate the M-INS’s enhanced accuracy, with SEP values witnessing a marked decrease from 7.23 m, 6.21 m, and 7.77 m to 2.29 m, 2.24 m, and 2.38 m, amounting to significant improvements of 68.33%, 63.93%, and 69.37%, respectively. This algorithm holds great potential, especially in fields such as autonomous driving, robotic exploration, and other areas requiring dynamic navigation. Future work will not only focus on optimizing the algorithm’s performance, particularly in terms of adaptability to rapid dynamic changes and complex environments, but also include extensive long-time testing to evaluate the system’s performance over extended periods. Additionally, efforts will be made to integrate the MINS algorithm with other sensor fusion technologies to achieve higher accuracy and robustness in robot positioning across various complex scenarios. Through ongoing research and technological innovation, we aim to provide more reliable navigation solutions for robots in their diverse future applications.

## Figures and Tables

**Table 1:** M-INS SEP results.

No.	Before Processing	After Processing	Improvement
1	7.23	2.29	68.33%
2	6.21	2.24	63.93%
3	7.77	2.38	69.37%

## Data Availability

The data presented in this study are available on request from the corresponding author.
